# Contrasting capabilities of two ungulate species to cope with extremes of aridity

**DOI:** 10.1038/s41598-021-83732-w

**Published:** 2021-02-18

**Authors:** Melinda Boyers, Francesca Parrini, Norman Owen-Smith, Barend F. N. Erasmus, Robyn S. Hetem

**Affiliations:** 1 Centre for African Ecology, School of Animal, Plant and Environmental Sciences, University of the Witwatersrand, Johannesburg, 2050 South Africa; 2 Global Change Institute, University of the Witwatersrand, Johannesburg, 2050 South Africa; 3 Brain Function Research Group, School of Physiology, University of the Witwatersrand, Johannesburg, 2050 South Africa; 4 School of Animal, Plant and Environmental Sciences, University of the Witwatersrand, Johannesburg, 2050 South Africa; 5 Faculty of Natural and Agricultural Sciences, University of Pretoria, Pretoria, 0028 South Africa

**Keywords:** Behavioural ecology, Climate-change ecology, Physiology

## Abstract

Southern Africa is expected to experience increased frequency and intensity of droughts through climate change, which will adversely affect mammalian herbivores. Using bio-loggers, we tested the expectation that wildebeest (*Connochaetes taurinus*), a grazer with high water-dependence, would be more sensitive to drought conditions than the arid-adapted gemsbok (*Oryx gazella gazella*). The study, conducted in the Kalahari, encompassed two hot-dry seasons with similar ambient temperatures but differing rainfall patterns during the preceding wet season. In the drier year both ungulates selected similar cooler microclimates, but wildebeest travelled larger distances than gemsbok, presumably in search of water. Body temperatures in both species reached lower daily minimums and higher daily maximums in the drier season but daily fluctuations were wider in wildebeest than in gemsbok. Lower daily minimum body temperatures displayed by wildebeest suggest that wildebeest were under greater nutritional stress than gemsbok. Moving large distances when water is scarce may have compromised the energy balance of the water dependent wildebeest, a trade-off likely to be exacerbated with future climate change.

## Introduction

Large mammals are particularly vulnerable to climate change^1^. Because their slow life history traits do not allow for rapid evolutionary adaptation in terms of genetic modification, they will need to rely on behavioural and physiological flexibility if they are to survive^2^ the hotter and more arid future predicted for much of Africa^3^. If they are unable to adapt to rapidly changing conditions by changing their behaviour, physiology, and/or morphology or tracking appropriate climates because of anthropogenic impediments, two-thirds of South Africa’s mammals may face extinction by 2050^4^. It is therefore imperative to understand the behavioural and physiological flexibility of mammals facing drought to measure their resilience, particularly for large mammals currently inhabiting semi-arid environments^5^ that may epitomise future conditions. The Kalahari region of Botswana represents a semi-arid savanna with a lack of surface water, yet it supports an abundance and diversity of large mammalian herbivores. These ungulates appear to cope with seasonal extremes in temperature and aridity by seeking shade and reducing daytime activity^6,7^ to reduce evaporative water losses. However, species may differ in their behavioural and physiological responses, as well as their resilience to aridity. Rymer et al.^5^ proposed that enhanced phenotypic flexibility could predict whether a species will cope with increasing aridity, while acknowledging that arid-adapted species may have specialized adaptations that reduce the need for behavioural and physiological flexibility.

Among the large ungulates inhabiting the Kalahari region, the arid-adapted gemsbok (or South African oryx; *Oryx gazella gazella*) appears to possess a number of fixed functional traits allowing it to conserve body water and cope with the dry conditions^8^. For example, an increased relative medullary thickness may promote the production of concentrated urine, and a relatively large surface area to volume ratio of the spiral and distal colon may allow for more water to be absorbed, reducing moisture loss in dung^9^. Strong selective pressures likely facilitated these specialized adaptations, such that gemsbok have shown little change in abundance in response to past drought conditions^10^. Blue wildebeest (*Connochaetes taurinus*) appear to be more water-dependent with fewer fixed functional traits to conserve body water than gemsbok^9^, perhaps increasing their reliance on flexible behavioural or physiological responses to maintain homeostasis (allostasis). For example, a reliance on water may favour the selection of highly efficient muscles to facilitate seasonal migration between regions with adequate forage and water^11^, thereby increasing behavioural flexibility. Despite these physiological and behavioural differences, our previous work showed that both wildebeest and gemsbok displayed similar body temperature profiles in a typical year^7^. Yet, the wildebeest population in the Kalahari region collapsed by 90% during the extreme 1982/3 drought when fences blocked their access to remaining sources of surface water^12^. The population has not since recovered.

We compared the physiological and behavioural flexibility of water-dependent wildebeest and arid-adapted gemsbok during the extremely dry conditions that prevailed in the first of two successive hot-dry seasons. We expected that, compared with gemsbok, wildebeest would be less resistant to drought conditions and be less able to maintain a stable body temperature (homeothermy). Body temperatures may become more variable both as a result of higher maximum 24 h body temperatures, if evaporative cooling is switched off when water deprivation coincides with heat exposure, and lower minimum 24 h body temperatures, if low energy reserves reduce metabolic rates. We further expected wildebeest to select cooler microclimates and spend a greater proportion of their time in these cooler shaded microclimates to reduce heat loads during the heat of the day, which may result in reduced daytime activity. With fewer fixed functional traits to conserve body water^8^, we expected that wildebeest would spend more time travelling to diminishing surface water, thereby increasing total 24 h activity compared to gemsbok, especially during a drought season compared to a non-drought hot and dry season.

## Materials and methods

### Study area

The study took place in the south-western Kalahari region of Botswana, known as the Bakgalagadi Schwelle (S 24.35°, E 20.62°), including the Botswana side of the Kgalagadi Transfrontier Park. The vegetation forms an open savanna, overlying deep sandy substrate with limited free-standing water. There is an intermittent river, Nossob river, in the south, ~ 80 km from the centre of the study area. A characteristic of this area is the highly mineralized, clay-rich depressions called pans, which retain water for variable periods after rain^6^. Air temperatures exceed 40 °C in summer and fall below 1 °C in winter^6^. Rainfall is seasonal but erratic, falling primarily during short-duration, high-intensity thunderstorms between November and April^6^. Mean annual rainfall in the Schwelle region ranges between 250 and 350 mm^13^.

### Climatic variables

A free-standing miniature black globe thermometer (“miniglobe”), identical to the collar miniglobe thermometer, was placed within the area used by the animals in direct sun, 1 m aboveground, and recorded temperature (°C) every hour (S 24.307°, E 20.745°; reference miniglobe). Dry-bulb air temperature (°C), wind speed (ms^−1^), and solar radiation (Wm^−2^) data were obtained from the Agricultural Research Council (ARC) weather station located at the Nossob campsite (S 25.4°, E 20.6°). Normalised Difference Vegetation Index (NDVI) (MODIS Terra 16-day) and local rainfall (mm; CHIRPS) data covering the study area (S 24.434°, E 20.293°) were obtained from Google Earth Engine^14^.

### Study species and data collection

In August 2013, eight individual female gemsbok and eight individual female wildebeest, each from separate herds, were darted by a veterinarian from a helicopter. Each dart consisted of Thiafentanil (gemsbok: 7–8 mg, wildebeest: 4–6 mg, Thianil, Kyron Laboratories, Johannesburg, South Africa), medetomidine hydrochloride (gemsbok: 3–6 mg, wildebeest: 2–4 mg, Kyron Laboratories, Johannesburg, South Africa) and ketamine (gemsbok: 75–150 mg, wildebeest: 50–150 mg Pfizer Animal Health, Sandton, South Africa). Each individual was fitted with a GPS collar (African Wildlife Tracking, Pretoria, South Africa) that supported a miniglobe attached to the top to record the thermal environment that the individual bearing it occupied^15^. Miniglobe temperatures and GPS locations were recorded hourly. In addition, each individual underwent surgery to implant miniature temperature-sensitive data loggers in the retroperitoneal space and had a motion-sensitive data logger tethered to the abdominal muscle wall (see^7^ for details). The data loggers were covered with biologically and chemically inert wax (Sasol, South Africa) and sterilised in instant sterilant (F10 Sterilant with rust inhibitor, Health and Hygiene (Pty) Ltd., Roodepoort, South Africa) before implantation. Once the individual animal was immobile, it was placed in sternal recumbency with its head elevated and supported by sandbags. Following intubation, anaesthesia was maintained with 2–5% isoflurane (Aerrane, Astra Zeneca, Johannesburg, South Africa), administered in 100% oxygen. Incision sites were shaved and sterilised with chlorhexidine gluconate (Hibitane, Zeneca, Johannesburg, South Africa). A local anaesthetic (3 ml subcutaneously (S.C.); lignocaine hydrochloride, Bayer Animal Health (Pty) Ltd., Isando, South Africa) was administered to the incision site. After placement of the loggers, the incision was sutured closed. Respiratory rate, heart rate, arterial oxygen saturation, and rectal temperature were monitored throughout the surgery, which lasted approximately 30–45 min. Each individual animal also received an antibiotic (~ 0.04 ml kg^−1^, intra muscularly (I.M.), Duplocillin, Schering-Plough Animal Health Ltd., New Zealand), and anti-inflammatory (~ 0.5 mg kg^−1^ I.M., Metacam, Meloxicam injectable solution, Boehringer Ingelheim Vetmedica, Inc, St. Joseph, U.S.A.) medication. Following surgery and termination of inhalation anaesthesia, the immobilization drugs were completely reversed by a combination of naltrexone (gemsbok: 75–120 mg, wildebeest: 60–100 mg, I.M. Naltrexone, Kyron Laboratories, Johannesburg, South Africa) and atipamezole (gemsbok: 10–20 mg; wildebeest: 10–15 mg, I.M. Antisedan, Orion Corporation, Orion Pharma, Finland).

**Figure 1:**
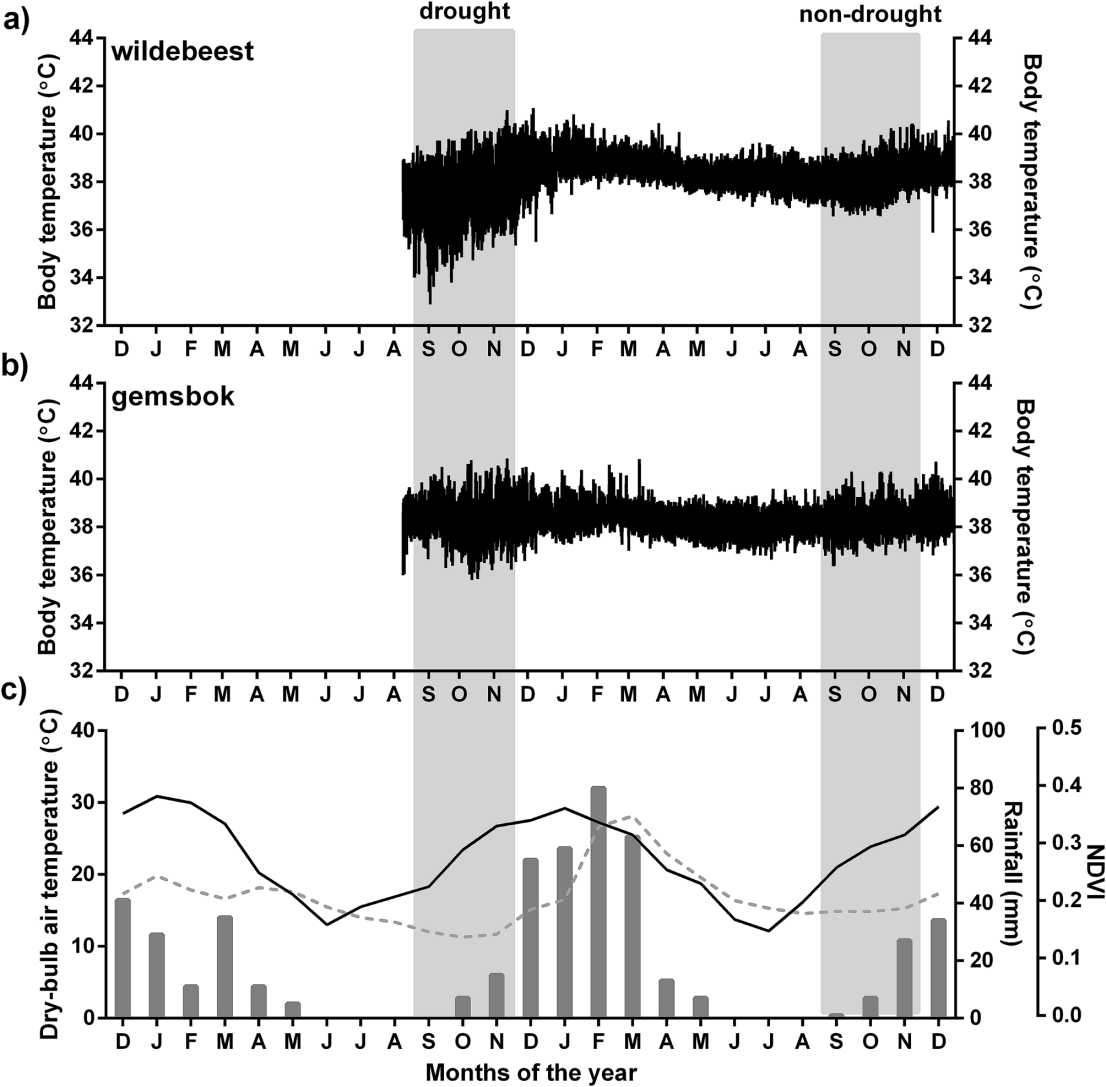
Ten-min recordings of body temperature from a representative female wildebeest (**a**) and female gemsbok (**b**) over the study period (September 2013 to November 2014); and the monthly dry-bulb air temperature (solid black line), rainfall (grey bars) and monthly composited vegetation greenness (NDVI; dashed grey line) over two years (**c**) highlighting drought conditions in the first year. The light grey boxes represent the two hot-dry seasons compared in the current study.

The temperature-sensitive data loggers (DST centi-T, Star-Oddi, Iceland) recorded body temperature at 10-min intervals (Fig. 1a,b) and the motion-sensitive data logger recorded whole body movements (i.e., motion changes) as activity counts within the first 10 s of each 5-min interval. The motion-sensitive logger had a triaxial accelerometer (ADXL345, Sigma Delta Technologies, Australia) with equal sensitivity across three planes (resolution one-fourth 4 mg/least significant bit). We adjusted the activity units to be relative to the maximum activity count for the entire study period per logger, to account for differences in the sensitivity of the individual motion-sensitive loggers. The data loggers and the collar weighed less than 1% of the individual animal’s body mass and is unlikely to have adversely affected their behaviour.

Two wildebeest were never relocated, possibly as a result of collar failure or predation. Three gemsbok died in October 2013. The remaining 11 animals were recaptured in May 2015, and data loggers and collars were removed. Thereafter the animals were released. Because of the inability to relocate all animals, animal deaths, and technological failures, we recovered a sample of 11 internal body temperature loggers (five gemsbok and six wildebeest); eight internal motion-sensitive loggers (four gemsboks and four wildebeest); nine GPS units (five gemsboks and four wildebeest) and nine miniglobe temperature sensors from the collars (five gemsboks and four wildebeest).

All procedures were approved by the Animal Ethics Screening Committee of the University of the Witwatersrand (protocol no. 2012/24/04) and all experiment procedures were performed in accordance with relevant guidelines and regulations as well as the ARRIVE guidelines (https://arriveguidelines.org/). The Government of Botswana via the Ministry of Environment, Wildlife and Tourism and Department of Wildlife and National Parks granted approvals and permits [numbers EWT 8/36/4 XX (32), EWT 8/36/4 XXVII (15), EWT 8/36/4 XXIV (102)] to conduct the study.

### Data analysis

During the study period, the first hot-dry season (September to November 2013, ‘drought’) occurred at the end of a prolonged dry period, whereas the second hot-dry season (September to November 2014, ‘non-drought’) followed more typical rainfall conditions (Fig. 1c). Miniglobe temperature (24 h mean, minimum and maximum) and dry-bulb air temperature (24 h minimum and maximum), as well as mean 24 h wind speed and solar radiation were similar between the two hot-dry periods (Table 1). We averaged 16-day NDVI composites per season as an index of vegetation greenness in response to prior rainfall. Rainfall during the wet season prior to the commencement of the study (December to May 2013) was less than 40% (< 132 mm) and outside of the 95% confidence interval of the long-term average (255 ± 56 mm between 1981 and 2017) for the study area^14^, whereas rainfall during the subsequent wet season (277 mm) was within the long-term range (Table 1). The lower rainfall preceding the drought period resulted in lower vegetation greenness (as indexed by NDVI) compared to the non-drought hot-dry season (Table 1; Fig. 1c).

**Table 1:** Environmental conditions (mean ± SD) that prevailed in the two hot-dry seasons over the study period within the Bakgalagadi Schwelle, Botswana.

	Drought (Sep – Nov 2013)	Non-drought (Sep – Nov 2014)
Miniglobe temperature (°C)		
24 h mean	24.8 ± 4.7	25.1 ± 2.5
24 h maximum	40.2 ± 5.5	42.1 ± 2.8
24 h minimum	10.6 ± 4.0	9.8 ± 1.9
Dry-bulb air temperature (°C)		
24 h maximum	34.9 ± 3.8	35.2 ± 1.6
24 h minimum	10.8 ± 4.7	11.7 ± 3.2
Mean 24 h solar radiation (Wm^−2^)	26.8 ± 4.1	24.4 ± 2.8
Mean 24 h wind speed (ms^−1^)	1.6 ± 0.1	1.7 ± 0.2
Total rainfall (mm) during season	22	35
Number of rainy days during season	6	11
NDVI (16-day composites)	0.146 ± 0.005	0.188 ± 0.003
Wet season (Dec – May) rainfall (mm) prior to hot-dry season	132	277

For consecutive 24 h periods, we calculated minimum, maximum and amplitude (maximum minus minimum) of the 24 h rhythm of body temperature. To determine behavioural adjustments in heat load brought about by microclimate usage, we calculated the difference between miniglobe temperature on the collar and the reference miniglobe per hour per individual animal. We calculated the time spent in microclimates cooler than direct sun by summing the hours when collar miniglobe was more than 0.5 °C lower than the reference miniglobe during daylight hours. We calculated a cumulative measure of cool microclimate usage during daylight hours per day as the sum of the hourly differences between collar miniglobe and reference miniglobe temperatures when animals were in cool microclimates (i.e., collar miniglobe was more than 0.5 °C lower than the reference miniglobe). To assess time spent travelling, we calculated the number of hours per day in which animals traversed more than 1.6 km within an hour, a distance previously associated with at least 50% of the hour engaged in directed movements at maximum walking speeds of ~ 3 km h^−1^^16,17^. We also calculated total 24 h activity and the proportion of activity that took place during the heat of the day (between 10:00 and 16:00) per individual. We used a series of Generalised Linear Mixed Models (GLMMs; Gaussian error family for continuous data, Binomial error family for proportions and Poisson error family for counts) to test the association between season and species in terms of body temperature profiles, cumulative measure of cool microclimate use and time spent in cool microclimates per day, time spent travelling per day, total 24 h activity and the proportion of activity that took place during the heat of the day (10:00 to 16:00). Model assumptions of residual normality and homoscedasticity were assessed graphically within R software^18^, informing subsequent data transformations and model choices. Season, species and an interaction between season and species were represented as fixed effects with individual animals and date included as random effects to control for repeated measures per individual per season. The response values of the body temperature profiles were heteroscedastic, so we included a variance structure and correlation structure within the models to correct for heteroscedasticity^19^. The total 24 h activity response values were log transformed for normality. We first fitted the global model, containing all the explanatory variables and interactions, then fitted season or species on their own. For example, our activity models took the form: log (total 24 h activity) ~ season + species + interaction between season and species + individual animals (random effect) + date (random effect). The coefficients reported are for the non-drought hot-dry season, wildebeest, and the interaction for wildebeest in the non-drought hot-dry season compared to the reference levels: drought, gemsbok, and gemsbok in the drought, respectively. Using model selection procedures of Akaike’s Information Criterion, corrected for small sample bias (AICc) and model weighting (*wi*)^20^, we established the importance of each fixed variable and identified which model best supported the data^21^. Of all the candidate models, all models with a difference in AICc value of < 2 were considered plausible and were used for making inferences^20^. We report on the effects size estimates and their precision (95% confidence intervals (CI)) per model as well as the conditional R^2^ for mixed-effect models. The conditional R^2^ describes the proportion of variance explained by both the fixed and random factors^22^. We used the R statistical software^18^ with R packages nlme^23^ and lme4^24^ to perform the GLMM analysis, AICcmodavg^25^ to perform the model selection, and emmeans^26^ to estimate marginal means from models. All graphs were created in GraphPad Prism (version 6.00 for Windows, GraphPad Software, San Diego, CA, USA).

## Results

### Body temperature

**Figure 2:**
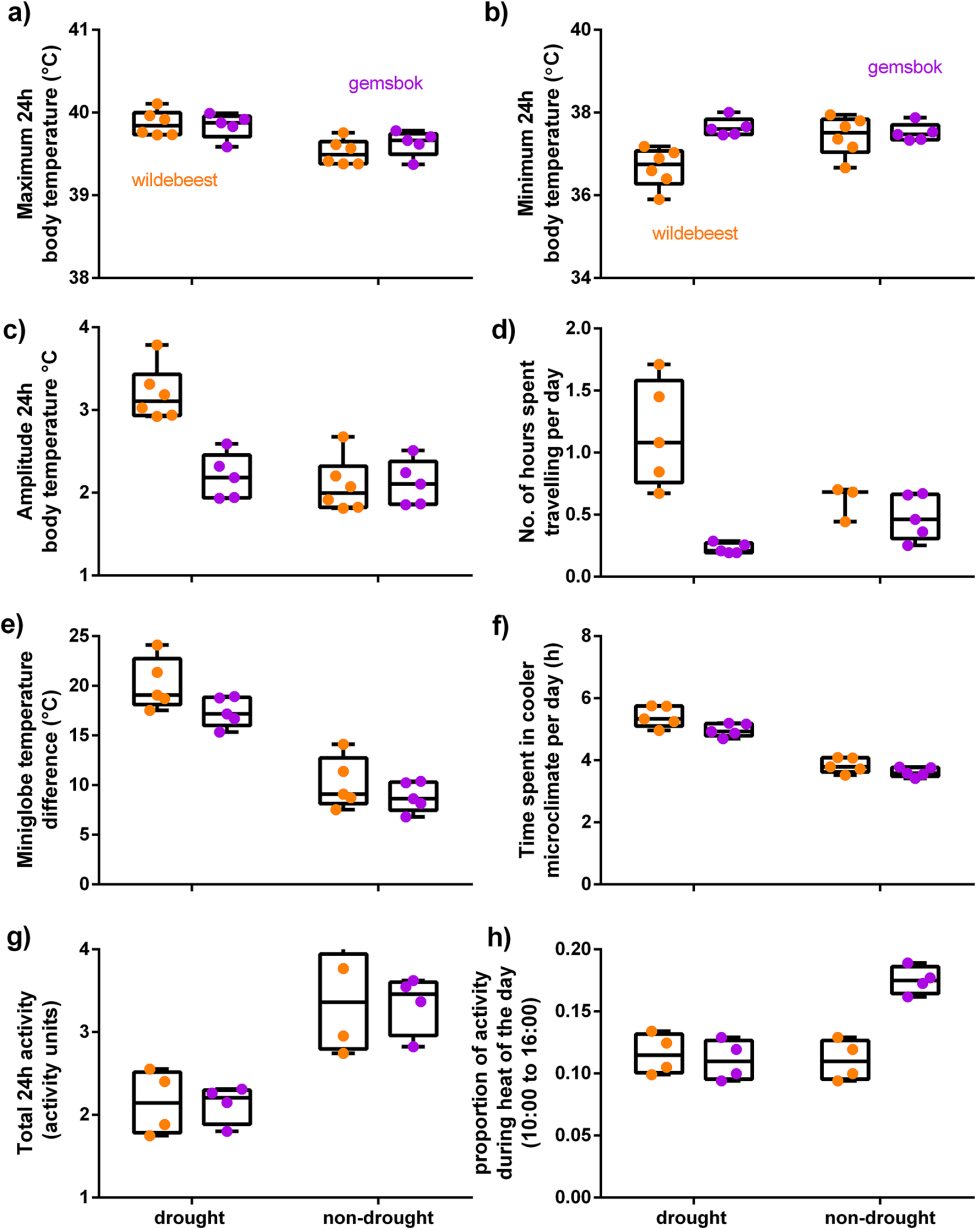
Predictive margins with 95% CIs for the Generalised Linear Mixed Model results for the association between season and species in terms of body temperature profiles (**a**–**c**), number of hours spent travelling per day (**d**), cumulative microclimate use (**e**) and time spent in cool microclimates (**f**) per day, total 24 h activity (**g**) and the proportion of activity that took place during the heat of the day (10:00 to 16:00) (**h**).

**Figure 3:**
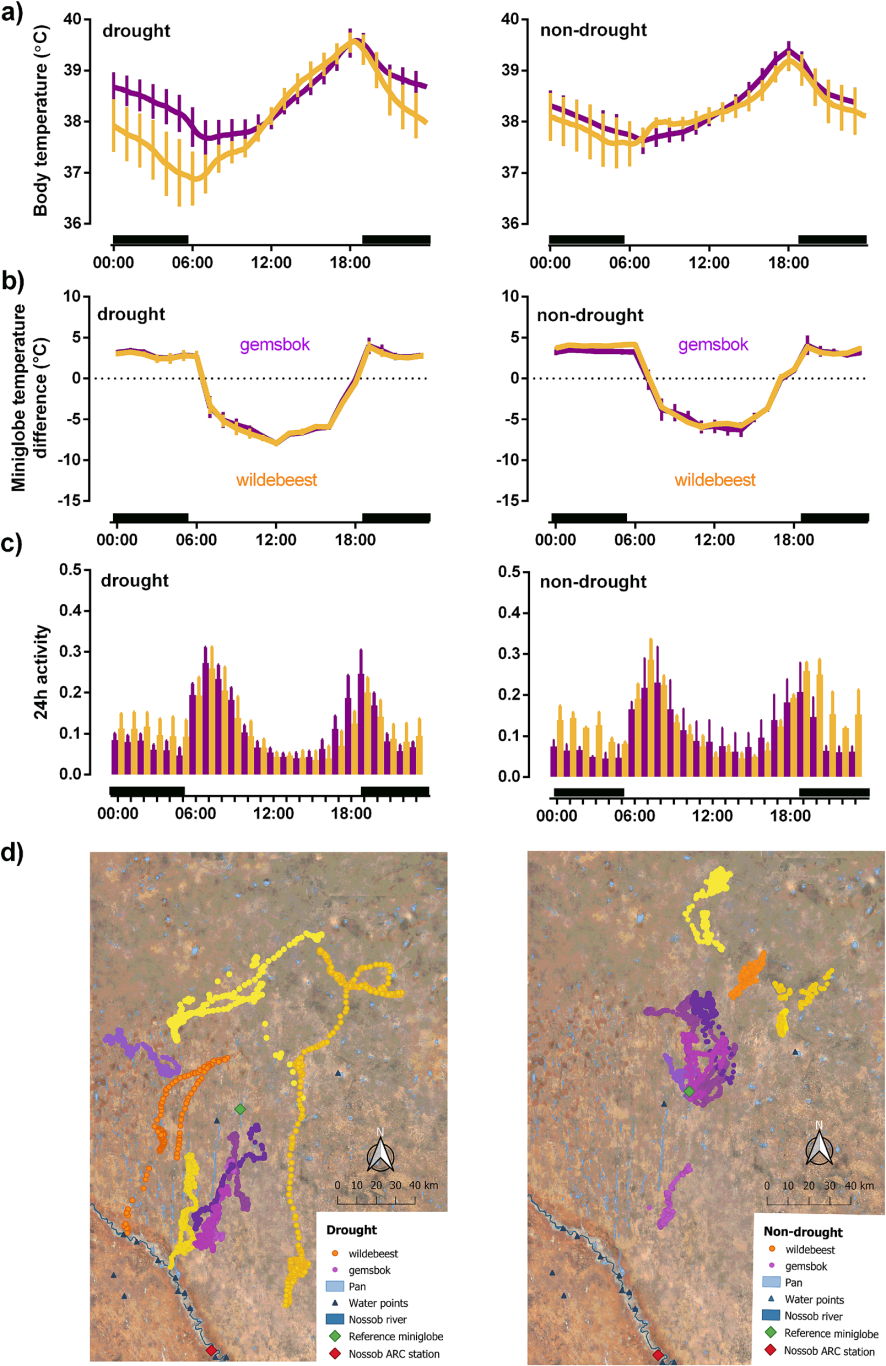
(**a**) 24-h rhythm (mean ± hourly SD) of body temperature for gemsbok (n = 5, purple line) and wildebeest (n = 6, orange line); (**b**) microclimate selection for gemsbok (n = 5, purple line) and wildebeest (n = 2, orange line); (**c**) activity for gemsboks (n = 3, purple bars) and wildebeest (n = 3, orange bars); (**d**) GPS locations for gemsbok (n = 5, shade of purple dots) and wildebeest (n = 4, shade of orange dots) comparing the hot dry season during the drought year with the same season during more non-drought conditions in the following year. Microclimate usage is expressed as the difference between miniglobe temperature on the collar (i.e., at the site chosen by each individual and the temperature of an identical miniglobe exposed to the sun at a nearby reference site). Activity counts were calculated relative to the maximum recorded for the logger throughout the study period. Black horizontal bars represent night. Maps (3d) were created in QGIS 3.14.16-Pi (http://qgis.osgeo.org) using Google Satellite Imagery (Map data @2020: Google, TerraMetrics).

Compared to the non-drought hot-dry season, wildebeest displayed a greater increase (0.4 °C vs. 0.2 °C on average, Table 2) in maximum 24 h body temperatures (Fig. 2a) and a greater decline (− 0.7 °C vs. + 0.1 °C on average) in minimum 24 h body temperatures (Fig. 2b) than gemsbok during the drought (Table 2). The higher maximum body temperatures and lower minimum body temperatures displayed by wildebeest resulted in a ten times larger increase (1 °C vs. 0.1 °C on average) in the 24 h amplitude of body temperature rhythms (Fig. 2c) compared to gemsbok between the non-drought and drought season (Figs. 1a,b, 3a, Table 2).

**Table 2:** Results of Generalised Linear Mixed Models comparing the 24 h body temperature parameters, cumulative microclimate use, time spent in cooler microclimates, total 24 h activity, proportion of activity during the heat of the day (10:00 to 16:00), and number of hours spent travelling per day for gemsbok and wildebeest during the hot-dry seasons of the two successive years, as well as the interaction effects. The estimates and confidence intervals (CI) reported are for the non-drought hot-dry season, wildebeest, and the interaction for wildebeest in the non-drought season compared to the reference levels: drought, gemsbok, and gemsbok in the drought. The presented models are the most parsimonious of a general model (Supplementary Table 1). Date and individual animal were included as random effects.

Predictors	Estimate	(CI)	Estimate	(CI)	Estimate	(CI)
	Maximum 24 h body temperature (N = 2002, n = 11)		Minimum 24 h body temperature (N = 2002, n = 11)		Amplitude 24 h body temperature (N = 2002, n = 11)	
Intercept	39.84	(39.69–39.99)	37.64	(37.32–37.96)	2.2	(1.93–2.46)
Season: non-drought	− 0.21	(− 0.30 to − 0.12)	− 0.13	(− 0.25 to − 0.01)	− 0.08	(− 0.21 to 0.05)
Species: wildebeest	0.03	(− 0.21 to 0.26)	− 0.97	(− 1.49 to − 0.46)	1	(0.58–1.42)
Interaction: wildebeest in the non-drought season	− 0.14	(− 0.27 to − 0.01)	0.90	(0.71–1.08)	− 1.03	(− 1.21 to − 0.85)

	Cumulative microclimate use (N = 1177, n = 10)		Time spent in cooler microclimate (N = 1168, n = 10)			
Intercept	17.38	(14.65–20.12)	5.16	(4.54 – 5.86)		
Season: non-drought	− 8.54	(− 11.28 to − 5.79)	0.72	(0.61–0.84)		
Species: wildebeest	2.78	(0.11–5.44)				
Interaction: wildebeest in the non-drought season	− 1.45	(− 2.88 to − 0.01)				

	Total 24 h activity (N = 1183, n = 8)		Proportion of activity during the heat (N = 1183, n = 8)		Hours spent travelling per day (N = 1326, n = 10)	
Intercept	0.89	(0.74–1.04)	0.13	(0.11–0.14)	0.06	(0.04–0.09)
Season: non-drought	− 0.07	(− 0.17 to 0.03)	0.05	(0.03–0.07)	1.57	(1.01–2.43)
Species: wildebeest	− 0.13	(− 0.34 to 0.07)	− 0.01	(− 0.03 to 0.01)	4.68	(3.02–7.27)
Interaction: wildebeest in the non-drought season	0.52	(0.40–0.64)	− 0.05	(− 0.08 to − 0.03)	0.37	(0.20–0.69)

### Microclimate usage

Both species increased the time spent in cool microclimates from ~ 3.5 to 5 h per day during the drought (Table 2, Fig. 2f). The strongest model only included a seasonal effect, but there was some support for an interaction between season and species. However, the inclusion of species within the model did not significantly improve the model’s performance (the R^2^ was the same) and was uninformative^27^. Both species doubled their cumulative cool microclimate use during the drought compared to the non-drought hot-dry season (Table 2, Fig. 2e). The strongest model included an interaction between season and species, in which wildebeest displayed a slightly greater increase in the cumulative cool microclimate use than gemsbok during the drought (Table 2, Fig. 2e).

### Activity and long-distance movement

Nearly 20% of the gemsbok total 24 h activity fell during the heat of the day (10:00 to 16:00) in the non-drought hot-dry season. But during the drought season, gemsbok reduced their proportion of activity during the heat of the day almost by half (18% vs. 13%, Fig. 2h). Wildebeest were generally less active during the heat of the day than gemsbok (~ 12% of their total 24 h activity). Unlike gemsbok, they did not show any further decrease in the proportion of activity that took place during the heat of the day during the drought (Table 2, Fig. 2h). Although the timing of wildebeest activity did not change much with the drought, their overall or total 24 h activity (Table 2, Fig. 2g) declined substantially more than that of the gemsbok (− 36% vs. + 5%) during the drought. Despite this decline in total 24 h activity, wildebeest spent twice as much time per day (1.2 h vs. 0.6 h) travelling more than 1.6 km within an hour during the drought season (Table 2, Fig. 2d) than the non-drought hot-dry season (Table 2; Fig. 3d). One individual traversed 80 km over 3 days to the Nossob river during the drought (Fig. 3d). Unlike wildebeest, gemsbok halved their time spent in long-distance movements (0.2 h vs 0.5 h) during the drought.

## Discussion

Water-dependent wildebeest coped less well than arid-adapted gemsbok during the extremely dry conditions that prevailed during a drought. Despite our small sample size and no measure of resilience in terms of fitness, we were able to detect physiological wellbeing in the form of body temperature variability. During the drought, compared to the non-drought hot-dry season, wildebeest displayed a greater increase in maximum 24 h body temperatures and a greater decline in minimum 24 h body temperatures compared with gemsbok, which resulted in a larger increase in 24 h amplitude of body temperature rhythm (Fig. 3a). Based on the slightly higher cumulative cool microclimate use despite similar time spent in these cool microclimates, wildebeest generally increased the intensity of cool microclimate use more than gemsbok during the drought. Use of cool microclimates coincided with reduced activity during the heat of the day (Fig. 3b,c), with gemsbok able to reduce the proportion of activity during the heat of the day without reducing total 24 h activity and halving the time spent moving long distance per day during the drought. Conversely, wildebeest displayed a substantial reduction in total 24 h activity during the drought, despite doubling the time spent moving long distances per day, presumably to access surface water as suggested by the long-distance movements to the Nossob river or better quality forage (Fig. 3d). When quality resources become depleted, movement may decline, as observed in our gemsbok, potentially as a result of devoting more time to digesting low quality forage^28^, or become more directed, as observed in our wildebeest, in search of remaining reserves of better quality forage or surface water^29^. Both gemsbok and wildebeest are predominantly grazers, but gemsbok are known to differ in their dietary preferences. Wildebeest primarily forage on short-sward grasses that are high in quality, but can survive on a less nutritious diet provided surface water is available^30^, and maintain their crude protein intake by shifting to some browse to overcome the forage resource bottleneck during a non-drought dry season^31^. Gemsbok are better able to shift their diet to forage on low-quality grasses, dicotyledons and water-rich succulent plants in dry seasons^32,33^. Essentially, during the drought, gemsbok were sedentary (reduced excursions) but retained 24 h activity (presumably foraging) whereas wildebeest increased excursions but reduced 24 h foraging potentially to conserve energy. Walking long distances increases energetic costs at a time when energy gain is compromised because of reduced quality and quantity of vegetation (as indexed by NDVI; Table 1), particularly during the drought.

High energetic costs of moving long distances in search of surface water or quality forage during resource limited periods may be alleviated somewhat by highly efficient muscles^11^. However, when conditions become particularly dry during a drought, wildebeest might be unable to recoup the energetic costs via their diet or fat reserves, resulting in mass die-offs of wildebeest^12^. We, therefore, suggest that the wildebeest experienced an energy deficit and were unable to maintain metabolic costs of homeothermy, as reflected by their low minimum 24 h body temperatures during the drought. This failure of homeothermy may reflect an allostatic overload. Allostatic overload occurs when the cumulative cost of stability (homeostasis) becomes unsustainable under stressful conditions and may result in pathophysiology^34^. Specifically, type 1 allostatic overload results from an energy deficit and may result in activation of emergency life-history stages^35^. Indeed, low minimum body temperatures are associated with reduced reproductive output^36^, ultimately reducing fitness and resilience if adverse conditions persist^37^. The severity of the energy deficit observed in the wildebeest in this study is highlighted by the low minimum 24 h body temperatures, which were ~ 1 °C lower than rumen temperatures observed in Alpine ibex (*Capra ibex ibex*^38^) and llamas (*Lama glama*^39^) living in extreme high altitude environments where air temperatures frequently drops below − 10 °C. Exposed to more extreme seasonal variation in resources, the Northern Hemisphere ungulates are able to buffer energy deficiencies during resource-limited periods by mobilising their fat reserves^40^. Conversely, African ungulates store only a small fraction of the body fat reserves of their northern counterparts^41^, which may make them more vulnerable to resource limited periods during extreme droughts. Perhaps large subcutaneous fat reserves would impede heat dissipation in African ungulates exposed to more tropical climates.

In addition to the pronounced reduction in minimum 24 h body temperature reflecting an energy deficit during the drought, wildebeest also showed a slightly larger increase in maximum 24 h body temperature than the gemsbok, which may reflect a body water deficit. When ambient temperatures exceed body temperatures, ungulates rely on evaporative cooling (sweating or panting) to maintain homeothermy but will prioritize body water conservation over homeothermy when water is limited^42^. The narrow 24 h body temperature rhythm maintained by gemsbok throughout this study was similar to that reported for free-living gemsbok with access to water^43,44^ and implies that the gemsbok in this study were not energetically or water stressed by the drought. Ungulates attempt to save body water behaviourally by selecting cool microclimates (shade-seeking) to reduce radiant heat loads, thereby reducing the need for evaporative cooling^45,46^, with shade-seeking being enhanced when water is limited^46^. Both the gemsbok and wildebeest in this study were more judicious in their selection of high-quality microclimates and spent longer in cooler microclimates during the drought, which resulted in a reduced activity during the heat of the day. Whereas the gemsbok compensated for the reduced diurnal activity by increasing activity in the cooler times of day so that total 24 h activity remained unchanged, wildebeest reduced their total 24 h activity without changing the proportional timing of that activity (i.e., proportion of nocturnal and daytime activity remained constant), perhaps because of nutritional stress or high predation risk at night^47,48^. Wildebeest in other environments also show a remarkably consistent rhythm of activity around dawn and dusk^49,50^. Shifting to nocturnal activity is predicted to increase resilience to hotter and drier environments, particularly in environments where water and shade are limited and nocturnal predation risk low^51^. For example, in the more extreme Arabian Desert both Arabian oryx (*Oryx leucoryx*) and sand gazelle (*Gazella subgutturosa marica*) became completely nocturnal and remained in shade throughout the day during hot and dry period^52^. In the Arabian Desert, and occasionally in the current study, air temperatures in shaded areas exceeded body temperatures of 38 °C, reducing the efficiency of dry heat loss mechanisms and increasing reliance on evaporative cooling to maintain homeothermy^53^. Like the Arabian ungulates^52^, the wildebeest appeared to prioritize body water conservation over homeothermy, and in addition, they were presumably forced to expend limited energy reserves to access increasingly scarce surface water.

Walking long distances to find surface water or better quality of forage may become a risky strategy in a hotter and drier future, where water sources are no longer reliable and fragmented landscapes block historic migration routes as suggested by the die-offs experienced during the drought of 1982/3^12^. To better predict resilience to drought before animals perish, we need a measure of the efficacy of behavioural and physiological plasticity. Using body temperature as an index of physiological wellbeing^54^, we have shown that enhanced fixed functional traits of the arid-adapted gemsbok may be a more sustainable response to drought than the reduced overall activity and traversing large distances in search of unreliable water sources observed in the water-dependent wildebeest. With future climatic conditions predicted to be hotter and drier with more extreme and more frequent droughts^3^, water-dependent species may be at increased risk of extirpation in semi-arid regions.

## Supplementary information

Supplementary information.

## Data Availability

The data that support the findings of this study are available in AfriMove repository, www.afrimove.org.
