# Supplementary material for: Contrasting capabilities of two ungulate species to cope with extremes of aridity
Source: Sci Rep. 2021 Feb 18;11:4216. doi: 10.1038/s41598-021-83732-w (PMC7893036; doi:10.1038/s41598-021-83732-w)
Supplement: Supplementary file 1 — Supplementary information. [file 41598_2021_83732_MOESM1_ESM.docx]

**Contrasting capabilities of two ungulate species to cope with extremes of aridity.**

Melinda Boyers^1,3^*, Francesca Parrini^1^, Norman Owen-Smith^1^, Barend F.N. Erasmus^2,5^, and Robyn S. Hetem^3,4^

^1^Centre of African Ecology, School of Animal, Plant and Environmental Sciences, University of the Witwatersrand, Wits 2050, South Africa.

^2^Global Change Institute, University of the Witwatersrand, Wits 2050, South Africa.

^3^Brain Function Research Group, School of Physiology, University of the Witwatersrand, Wits 2050, South Africa.

^4^School of Animal, Plant and Environmental Sciences, University of the Witwatersrand, Wits 2050, South Africa.

^5^Faculty of Natural and Agricultural Sciences, University of Pretoria, Pretoria 0028, South Africa

* address for correspondence (email: boyers21@gmail.com)

**Supplementary Table 1.** Summary of the model ranking using Akaike Information Criterion (AICc) to test the variables affecting body temperature parameters, cumulative shade use, total 24h activity, proportion of activity during daylight hours, and no. of hours spent travelling per day for gemsbok and wildebeest during the hot-dry seasons of the two successive years. The coefficients reported are for the typical hot-dry season, wildebeest, and the interaction for wildebeest in the typical hot-dry season compared to the reference levels: drought, gemsbok, and gemsbok in the drought. Date and individual animal were included as random effects. The total 24h activity response values were log transformed for normality. *Di* is the delta weight (difference between the AIC for a given model and the best fitting model); *Wi* is the model selection probability (Akaike weights). The model with the lowest AICc is in bold.

|  | **Model 1** | | **Model 2** | | **Model 3** | | **Null model** | |
| --- | --- | --- | --- | --- | --- | --- | --- | --- |
| *Predictors* | *Estimates* | *CI* | *Estimates* | *CI* | *Estimates* | *CI* | *Estimates* | *CI* |
| **Minimum 24h body temperature (N = 2002, n = 11)** | | | | | | | | |
| Intercept | 37.64 | 37.32 – 37.96 | 37.11 | 36.85 – 37.37 | 37.58 | 37.26 – 37.89 | 37.29 | 37.02 – 37.56 |
| Season | -0.13 | -0.25 – -0.01 | -0.36 | -0.48 – -0.24 |  |  |  |  |
| Species | -0.97 | -1.49 – -0.46 |  |  | -0.53 | -1.03 – -0.03 |  |  |
| Interaction | 0.9 | 0.71 – 1.08 |  |  |  |  |  |  |
| Conditional R^2^ | 0.40 | | 0.34 | | 0.32 | | 0.35 | |
| AICc | **1530.7** | | 1685.5 | | 1722.7 | | 1777.6 | |
| Di | 0 | | 154.84 | | 192 | | 246.95 | |
| *Wi* | 1 | | 0 | | 0 | | 0 | |
| **Maximum 24h body temperature (N = 2002, n = 11)** | | | | | | | | |
| Intercept | 39.84 | 39.69 – 39.99 | 39.86 | 39.75 – 39.96 | 39.73 | 39.59 – 39.88 | 39.73 | 39.59 – 39.88 |
| Season | -0.21 | -0.30 – -0.12 | -0.29 | -0.35 – -0.22 |  |  |  |  |
| Species | 0.03 | -0.21 – 0.26 |  |  | 0.04 | -0.26 – 0.18 |  |  |
| Interaction | -0.14 | -0.27 – -0.01 |  |  |  |  |  |  |
| Conditional R^2^ | 0.1 | | 0.11 | | 0.07 | | 0.08 | |
| AICc | **2479.4** | | 2487.6 | | 2657.9 | | 2667.1 | |
| Di | 0 | | 8.23 | | 178.54 | | 187.72 | |
| *Wi* | 0.98 | | 0.02 | | 0 | | 0 | |
| **Amplitude 24h body temperature (N = 2002, n = 11)** | | | | | | | | |
| Intercept | 2.2 | 1.93 – 2.46 | 2.74 | 2.53 – 2.95 | 2.15 | 1.90 – 2.41 | 2.42 | 2.19 – 2.64 |
| Season | -0.08 | -0.21 – 0.05 | -0.64 | -0.76 – -0.53 |  |  |  |  |
| Species | 1 | 0.58 – 1.42 |  |  | 0.49 | 0.08 – 0.89 |  |  |
| Interaction | -1.03 | -1.21 – -0.85 |  |  |  |  |  |  |
| Conditional R^2^ | 0.37 | | 0.27 | | 0.18 | | 0.19 | |
| AICc | **3115.3** | | 3218.7 | | 3378.8 | | 3397.4 | |
| Di | 0 | | 103.43 | | 263.5 | | 282.13 | |
| *Wi* | 1 | | 0 | | 0 | | 0 | |
| **Cumulative microclimate use (N = 1177, n = 10)** | | | | | | | | |
| Intercept | 17.38 | 14.65 – 20.12 | 18.74 | 16.23 – 21.26 | 12.42 | 10.02 – 14.83 | 13.58 | 11.44 – 15.71 |
| Season | -8.54 | -11.28 – -5.79 | -9.03 | -11.74 – -6.32 |  |  |  |  |
| Species | 2.78 | 0.11 – 5.44 |  |  | 2.34 | -0.38 – 5.06 |  |  |
| Interaction | -1.45 | -2.88 – -0.01 |  |  |  |  |  |  |
| Conditional R^2^ | 0.76 | | 0.76 | | 0.75 | | 0.75 | |
| AICc | **7834.8** | | 7836.9 | | 7872.3 | | 7872.8 | |
| Di | 0 | | 2.19 | | 37.53 | | 38 | |
| *Wi* | 0.75 | | 0.25 | | 0 | | 0 | |
| **Time spent in cooler microclimate per day (N = 1168, n = 10)** | | | | | | | | |
| Intercept | 4.96 | 4.33 – 5.67 | 5.16 | 4.54 – 5.86 | 4.11 | 3.71 – 4.56 | 4.27 | 3.88 – 4.71 |
| Season | 0.73 | 0.62 – 0.86 | 0.72 | 0.61 – 0.84 |  |  |  |  |
| Species | 1.09 | 0.98 – 1.20 |  |  | 1.09 | 0.99 – 1.19 |  |  |
| Interaction | 0.98 | 0.87 – 1.09 |  |  |  |  |  |  |
| Conditional R^2^ | 0.54 | | 0.54 | | 0.55 | | 0.55 | |
| AICc | 4919.0 | | **4917.3** | | 4930.1 | | 4930.6 | |
| Di | 1.7 | | 0 | | 12.83 | | 0 | |
| *Wi* | 0.3 | | 0.7 | | 13.29 | | 0 | |
| **Total 24h activity (N = 1183, n = 8)** | | | | | | | | |
| Intercept | 0.89 | 0.74 – 1.04 | 0.82 | 0.73 – 0.91 | 0.87 | 0.76 – 0.97 | 0.87 | 0.80 – 0.95 |
| Season | -0.07 | -0.17 – 0.03 | -0.14 | -0.22 – -0.05 |  |  |  |  |
| Species | -0.13 | -0.34 – 0.07 |  |  | -0.01 | -0.13 – 0.16 |  |  |
| Interaction | 0.52 | 0.40 – 0.64 |  |  |  |  |  |  |
| Conditional R^2^ | 0.29 | | 0.21 | | 0.2 | | 0.2 | |
| AICc | **1701.8** | | 1765.6 | | 1775.0 | | 1773.0 | |
| Di | 0 | | 63.89 | | 71.26 | | 73.24 | |
| *Wi* | 1 | | 0 | | 0 | | 0 | |
| **Proportion of activity during heat of the day (10:00 - 16:00) (N = 1183, n = 8)** | | | | | | | | |
| Intercept | 0.13 | 0.11 – 0.14 | 0.12 | 0.10 – 0.14 | 0.15 | 0.13 – 0.16 | 0.13 | 0.12 – 0.15 |
| Season | 0.05 | 0.03 – 0.07 | 0.03 | 0.01 – 0.05 |  |  |  |  |
| Species | -0.01 | -0.03 – 0.01 |  |  | -0.03 | -0.05 – -0.01 |  |  |
| Interaction | -0.05 | -0.08 – -0.03 |  |  |  |  |  |  |
| Conditional R^2^ | 0.27 | | 0.25 | | 0.25 | | 0.25 | |
| AICc | -**2139.2** | | -2117.6 | | -2113.2 | | -2110.6 | |
| Di | 0 | | 25.98 | | 21.61 | | 28.57 | |
| *Wi* | 1 | | 0 | | 0 | | 0 | |
| **Hours spent travelling per day (N = 1326, n = 10)** | | | | | | | | |
| Intercept | 0.06 | 0.04 – 0.09 | 0.12 | 0.09 – 0.17 | 0.07 | 0.05 – 0.10 | 0.11 | 0.08 – 0.14 |
| Season | 1.57 | 1.01 – 2.43 | 0.78 | 0.57 – 1.07 |  |  |  |  |
| Species | 4.68 | 3.02 – 7.27 |  |  | 2.90 | 2.14 – 3.95 |  |  |
| Interaction | 0.37 | 0.20 – 0.69 |  |  |  |  |  |  |
| Conditional R^2^ | 0.04 | | 0.002 | | 0.038 | | 0.000 | |
| AICc | **2414.2** | | 2464.2 | | 2420.1 | | 2464.6 | |
| Di | 0 | | 5.92 | | 50.06 | | 50.41 | |
| *Wi* | 0.95 | | 0.05 | | 0 | | 0 | |
